# BRCA Testing for Patients Treated in Italy: A National Survey of Breast Centers Associated with Senonetwork

**DOI:** 10.3390/curroncol31070282

**Published:** 2024-06-30

**Authors:** Corrado Tinterri, Damiano Gentile, Francesco Caruso, Laura Cortesi, Michelino De Laurentiis, Lucio Fortunato, Donatella Santini, Daniela Turchetti, Alberta Ferrari, Alberto Zambelli, Senonetwork Italia Breast Centre Responders

**Affiliations:** 1 Breast Unit, IRCCS Humanitas Research Hospital, Via Manzoni 56, 20089 Milan, Italy; corrado.tinterri@hunimed.eu; 2 Department of Biomedical Sciences, Humanitas University, Via Rita Levi Montalcini 4, 20072 Milan, Italy; alberto.zambelli@hunimed.eu; 3 HICC Humanitas Istituto Clinico Catanese, 95045 Misterbianco, Italy; francesco.caruso@humanitascatania.it; 4 Azienda Ospedaliera-Universitaria di Modena, Università degli Studi di Modena e Reggio Emilia, 41121 Modena, Italy; hbc@unimore.it; 5 Dipartimento di Oncologia Senologica, Istituto Nazionale Tumori “Fondazione PASCALE”, 80131 Napoli, Italy; m.delaurentiis@istitutotumori.na.it; 6 Breast Centre, Azienda Ospedaliera San Giovanni-Addolorata, 00184 Rome, Italy; lfortunato@hsangiovanni.roma.it; 7 IRCCS Azienda Ospedaliero, Universitaria di Bologna Policlinico di Sant’Orsola, 40138 Bologna, Italy; donatella.santini@aosp.bo.it; 8 Department of Medical and Surgical Sciences, University of Bologna, 40138 Bologna, Italy; daniela.turchetti@unibo.it; 9 Medical Genetics Unit, IRCCS Azienda Ospedaliero-Universitaria di Bologna, 40138 Bologna, Italy; 10 Fondazione IRCCS Policlinico San Matteo di Pavia, 27100 Pavia, Italy; a.ferrari@smatteo.pv.it; 11 Medical Oncology and Hematology Unit, IRCCS Humanitas Research Hospital, Via Manzoni 56, 20089 Milan, Italy

**Keywords:** breast cancer, BRCA testing, genetic counseling

## Abstract

Background: Breast units (BUs) provide breast cancer (BC) care, including prevention, treatment, and genetic assessment. Genetic research has highlighted BRCA1/2 mutations as key hereditary BC risk factors. BRCA testing is crucial for personalized treatment and prevention strategies. However, the integration of BRCA testing in Italian BUs faces multiple challenges. This study, by Senonetwork Italia, aimed to evaluate genetic testing practices and identify obstacles within Italian BUs. Methods: Senonetwork Italia conducted a 16-question web-based survey involving 153 BUs. The survey assessed aspects of BRCA testing, including timing, urgency, counseling, patient selection, and multi-gene panels. Results: Of the 153 BUs, 109 (71.2%) responded. Testing before surgery was performed by 70.6% of centers, with urgent cases acknowledged by 87.2%. Most centers (56.0%) arranged urgent pre-test counseling within a week. BRCA mutation status influenced treatment decisions in 99.1% of cases. Multi-gene panels were used by 33.0% of centers for all genetic counseling cases, while 56.0% followed standard referral criteria. The main challenges included cost, reimbursement, and reporting timelines. Conclusions: This survey highlights significant variations in BRCA testing practices across Italian BUs and identifies key logistical and financial challenges. There is a need for standardized practices of genetic testing to ensure personalized and effective BC management in Italy.

## 1. Introduction

A Breast Unit (BU) is defined as a facility dedicated to delivering comprehensive breast care services on a multidisciplinary basis. These services include breast cancer (BC) prevention and genetic assessment, the treatment of the primary tumor, accurate cancer surveillance, and the management of metastatic disease, along with supportive, palliative, and survivorship care [1,2]. Over the past 25 years, substantial evidence has indicated that BC patients treated in specialized BUs by dedicated specialists exhibit improved long-term oncological outcomes, contributing to a significant survival advantage [3,4,5]. In December 2014, the Italian Ministry of Health issued a directive [6] to the regional Departments of Health (responsible for healthcare delivery) mandating the establishment of regional networks of BUs. Each unit is required to manage a minimum of 150 new BC cases annually and adhere to the technical criteria set by the European Society of BC Specialists (EUSOMA) [7]. In recent years, advances in genetic research have identified mutations in BRCA1 and BRCA2 genes as key contributors to inherited BC risk. The utilization of BRCA genetic testing has emerged as a crucial tool in the proactive management of BC. By identifying individuals carrying BRCA mutations, healthcare providers can implement targeted preventive measures and personalized treatment strategies [8,9]. In this context, the strategic application of BRCA testing in understanding the genetic landscape of BC aids in risk stratification, which is crucial for identifying individuals with high risk who may benefit from intensified surveillance or risk-reducing interventions [10,11,12,13]. This holds profound implications for both prevention and early intervention since BC still remains the leading cause of mortality among women worldwide [14,15]. Moreover, knowledge of BRCA status plays a critical role in therapeutic decisions, guiding the selection of tailored treatments such as poly(ADP–ribose) polymerase (PARP) inhibitors for those individuals with pathogenic BRCA mutations [16,17,18,19,20]. However, the integration of BRCA testing into the framework of Italian BUs has to face several challenges, including limited access to genetic testing, availability of genetic counseling, constraints of regional guidelines, and ultimately, inequities in care access across different geographical areas. For these reasons, there is an urgent need to better integrate genetic testing for hereditary BC risk into primary care settings in Italy. As a cornerstone of future preventive medicine, genetic testing remains a largely untapped resource for patients who could benefit from additional cancer surveillance or riskreduction strategies. The clinical impact of this testing depends on the successful identification of individuals who are at risk, effective testing delivery, and diligent follow-up care for individuals with positive results. Although various interventions have been implemented, they have achieved mixed success in boosting hereditary risk assessment and genetic testing uptake due to barriers at the patient, provider, and system levels [21]. Despite these challenges, the Senonetwork Italia project prioritizes the incorporation of BRCA testing into clinical practice to offer a tangible opportunity for more effective and personalized treatments. Little is known regarding the real-world implementation of genetic testing at a national level for women diagnosed with BC; for these reasons, Senonetwork Italia promoted a web-based survey to examine the prevailing approach to genetic testing within primary care environments, identify the multifaceted obstacles to its wider application, and evaluate the effectiveness of existing strategies among BUs in Italy.

## 2. Materials and Methods

The Senonetwork Italia project was established to promote collaboration and information exchange among BUs, with the aim of improving the diagnosis and treatment of breast pathology in Italy’s dedicated centers that comply with the national standard requirements. Membership in Senonetwork is voluntary, and requires meeting access criteria in terms of the number of new cases/year and self-declaring compliance with quality requirements. Currently, 156 BUs are affiliated, accounting for more than 95% of women who are treated every year in Italy for BC. The project facilitates collaboration between BUs, ensures quality control within centers, promotes specialist training, supports clinical research, and initiates educational programs for women. In an effort to assess the therapeutic role of BRCA testing, Senonetwork Italia developed a comprehensive 16-question survey. Questions were prepared and agreed upon by a multidisciplinary Steering Committee during several briefings held online prior to the invitation to participate in the survey. The national survey was performed via a web-based platform between 1 June and 22 August 2023. The invitation to participate was sent via e-mail to the coordinators of various BUs. The clinical directors from each center participated by anonymously completing the questionnaire electronically, with no financial incentives provided for participation. The processing and analysis of data were managed by the secretariat of Senonetwork Italia. The findings from the survey were subsequently presented at the National “Attualità in Senologia” (AIS) Biannual Congress, held in Florence from 27 to 29 September 2023. The primary aim of the survey was to evaluate several key aspects of BRCA testing within clinical settings. These included the timing and urgency of testing, the implementation of pre-test counseling, patient selection based on BC subtypes, and the clinical implications of BRCA variants on treatment decisions. Additionally, the survey explored the comparative usage of multi-gene panels versus single-gene testing, as well as the integration of somatic-to-germline pathway assessments to accelerate genetic evaluations. The structure of the survey grouped questions into various domains, covering the following: the identification of BUs, access to and timing of genetic tests, challenges encountered in administering genetic tests, current practices in genetic counseling, criteria for selecting patients for testing, the utilization of multi-gene panels, and the application of somatic genetic assessments for therapeutic decision-making. Given that the survey was conducted anonymously and did not involve the analysis of patient data, the need for institutional review board approval was waived.

## 3. Results

Among the 153 BUs associated with Senonetwork Italia as of 1 June 2023, 109 (71.2%) responded to the survey on the therapeutic role of BRCA testing. The responses were collected from BUs across Italy, with 64 centers (58.7%) located in northern Italy, 26 centers (23.9%) in central Italy, and 19 centers (17.4%) in southern Italy/islands. Regional representation was led by Lombardy (22.0%). In terms of caseload, 27 centers (24.8%) managed 150–200 cases annually, 25 centers (22.9%) handled 201–300 cases, 21 centers (19.3%) had 301–400 cases, and 36 centers (33.0%) managed over 400 cases. Regarding the access and timing of the BRCA test, 70.6% of centers performed the test before surgery, 24.8% during early-stage treatment, and 4.6% at metastatic recurrence. Regarding the need for urgent testing, the majority of BUs (87.2%) acknowledged such circumstances. For urgent pre-test genetic counseling, including the mainstreamconsent approach (i.e., mini-counseling), 61 centers (56.0%) arrange the counseling within a week, 38 centers (34.9%) within 2–3 weeks, and 10 centers (9.1%) in over three weeks. Regarding test execution and clinical reporting times in urgent cases, 55.1% reported a turnaround period from the submission of the patient’s blood and the delivery of the test result of less than 21 days, 44.0% between 21 and 40 days, and 0.9% between 40 and 60 days. The logistics and timelines of BRCA testing are summarized in Table 1.

Question 8 reported that the majority of the analyzed BUs face challenges in BRCA testing procedures (53.2%), including obstacles for cost and reimbursement (11.9%), reporting timelines (35.7%), and pre-test counseling availability (13.8%). In the majority of BUs (78.9%), the mainstream-consent approach for genetic counseling is routinely conducted, usually led by medical oncologists (85.3%) and breast surgeons (67.9%). Regarding the prevalence of genetic tests by BC subtypes, it has been observed that for triple-negative BC patients, 53 BUs (48.6%) tested all patients, while the others tested according to age: 30 centers (27.5%) under the age of 60 and 23 centers (21.1%) under 50. For hormone-receptor-positive BC patients, 97 BUs (89.0%) initiated BRCA testing for those with early-onset disease (age < 36 years old), multiple or bilateral tumors, or a family history suggestive of hereditary BC risk. Regarding the possible expansion of BRCA assessment in accordance with the criteria for PARP inhibitor eligibility, the majority of BUs (*n* = 94, 86.2%) endorsed genetic testing in all patients potentially eligible for PARP inhibitors, regardless of the BC setting or subtypes, while a minority (*n* = 3, 2.8%) found current genetic counseling criteria sufficient without the need for an extended testing use eventually led by the therapeutic options. Question 15 reported the frequency of multi-gene panel usage within the included BUs, with 36 centers (33.0%) using multi-gene panels in all BC patients initiated to genetic counseling and 9 centers (8.3%) applying them in all patients potentially eligible for PARP inhibitor treatment. Meanwhile, 61 centers (56.0%) found that the standard criteria for referral to genetic counseling were sufficient, and only 3 centers (2.7%) reported never using multi-gene panels. The presence of a germline pathogenic BRCA variant influenced loco-regional treatment decisions in 108 centers (99.1%), and 102 centers (93.6%) believed somatic variants on tumor tissue would be of some utility for therapeutic purposes. The challenges and practices of BRCA testing are summarized in Table 2.

## 4. Discussion

This survey is the first national inquiry in Italy studying various aspects of BRCA testing. Notably, the survey involved 33.0% of centers managing over 400 cases of malignant breast pathology annually. This high volume signifies that a substantial number of BC patients at these centers could be eligible for genetic testing. According to the guidelines from the Society of Surgical Oncology (ASCO), all patients newly diagnosed with BC at stages I-III or with de novo stage IV/metastatic disease who are 65 years or younger should be offered BRCA1/2 testing (Formal Consensus; Agreement: 87.5%) [22]. Furthermore, patients over 65 should also receive testing if they are candidates for PARP inhibitor therapy, have triple-negative BC, have a personal or familial history indicating a possible pathogenic variant, were assigned male sex at birth, or belong to populations with a heightened prevalence of founder mutations, such as Ashkenazi Jewish ancestry (Formal Consensus; Agreement: 92.50%) [22]. Additionally, ASCO recommends that patients undergoing BRCA1/2 testing should be considered for additional testing for other cancer predisposition genes based on their personal or family history, with consultation from providers skilled in clinical cancer genetics to aid in decision-making (Formal Consensus; Agreement: 90%) [22]. However, while ASCO guidelines suggest a broad criterion for genetic testing, the actual practice in Italian BUs, as evidenced by our survey, shows a more selective approach: 48.6% of centers test all patients with triple-negative BC, and 89.0% of hormone-receptor-positive BC patients are initiated for BRCA testing primarily when they present with early-onset disease, multiple tumors, or a family history indicative of a genetic predisposition, highlighting a more tailored strategy that addresses specific patient profiles and risk factors.

The timing of BRCA testing was a focal point of the survey, revealing that a significant proportion of BUs perform the test at the diagnosis or pre-surgical stage, reflecting a timely approach that is consistent with the observed increased preference for risk-reducing mastectomy (RRM) when BRCA mutation status is known before surgery. Furthermore, the presence of a germline pathogenic BRCA variant almost invariably influenced loco-regional treatment decisions and overall treatment strategies (99.1%). Woo J et al. [23] investigated this issue, conducting a retrospective review of BC patients tested for BRCA1/2 mutations between January 2008 and November 2019 who underwent surgery at a single institution in South Korea. Among the 344 eligible patients, 140 (40.7%) were aware of their BRCA1/2 mutation status prior to surgery, while 204 (59.3%) discovered their status post-surgery. The timing of genetic diagnosis had a profound impact on the choice of surgery. Notably, the contralateral RRM rate was significantly higher among patients who were aware of their mutation status before surgery (45.0%, 63/140), compared to a minority of patients (2.0%, 4/204) diagnosed after surgery (*p*< 0.001). Additionally, the results from our survey revealed that the majority of centers (87.2%) recognized the importance of urgent testing in specific clinical situations. For urgent pre-test genetic counseling, many centers (56.0%) arrange it within a week, while test execution and reporting times show that the majority (55.1%) achieve a turnaround in less than 21 days. Similarly, it has been reported that the time interval between the submission of the patient’s peripheral blood and delivery of the verified test result can vary, averaging 21 days for those diagnosed pre-surgery versus 29 days post-surgery (*p*< 0.001) [23]. 

Several challenges in BRCA testing were identified by our survey, including cost and reimbursement issues (11.9%) and variations in reporting timelines (35.7%). Similar issues were reported by Dusic EJ et al. [21], who critically evaluated the landscape of genetic testing, identifying multiple barriers that hinder its effective implementation. Their study showed that current primary care models for genetic testing are inefficient, placing undue burdens on providers and patients and resulting in low uptake and underutilization of genetic services. Furthermore, despite various interventions aimed at increasing genetic testing uptake, their efficacy remains limited due to these pervasive obstacles. The authors recommended a significant shift towards integrating genetic testing into routine medical care via population-based approaches, proposing that this could significantly mitigate existing challenges and facilitate broader access to these services [21]. We believe that these challenges underscore the need to address logistical and financial considerations to enhance the integration of BRCA testing into clinical practice.

The results from our survey showed that while a significant proportion of BUs adhered to standard genetic counseling referral criteria for hereditary risk, a notable percentage (86.2%) of centers opted to test all patients potentially eligible for PARP inhibitors. This approach aligns with several studies [24,25,26,27,28] that inform the question of the role of BRCA1/2 testing to guide the use of PARP inhibitors in the treatment of patients with human epidermal growth factor receptor 2 (HER2)–negative BC. The EMBRACA trial [24], published in 2018, investigated the efficacy and safety of the PARP inhibitor talazoparib in treating advanced BC patients with a germline BRCA1/2 mutation. This randomized, open-label, phase III trial compared talazoparib (287 participants) to standard single-agent chemotherapy (144 participants), which included choices like capecitabine, eribulin, gemcitabine, or vinorelbine. The results demonstrated a notable improvement in median progression-free survival (PFS) for the talazoparib group, which was 8.6 months compared to 5.6 months in the standard chemotherapy group. This difference was statistically significant, with a hazard ratio (HR) for disease progression or death at 0.54 [95% confidence interval (95 %CI), 0.41–0.71; *p*< 0.001]. Talazoparib also showed superior patient-reported outcomes, significantly delaying the time to clinically meaningful deterioration in global health status and quality of life compared to standard chemotherapy. The OlympiAD trial, conducted by Robson M et al. [25], assessed the efficacy and safety of olaparib, another PARP inhibitor, compared to standard single-agent chemotherapy in women with HER2-negative metastatic BC who also had a germline BRCA1/2 mutation. In this randomized, open-label, phase 3 trial, 205 patients received olaparib and 91 received standard chemotherapy (capecitabine, eribulin, or vinorelbine). The primary endpoint was PFS, which was significantly longer in the olaparib group at a median of 7 months compared to 4.2 months in the chemotherapy group, with an HR for disease progression or death of 0.58 (95% CI, 0.43–0.80; *p*< 0.001). Olaparib also demonstrated a higher response rate at 59.9% compared to 28.8% in the chemotherapy group. Tutt ANJ et al. [26] reported that one year of adjuvant olaparib following the completion of local treatment and neoadjuvant or adjuvant chemotherapy led to a significant improvement in invasive disease-free survival (DFS) and distant DFS of patients with HER2-negative early BC harboring BRCA1 or BRCA2 germline pathogenic variants, who had high-risk clinicopathological factors. The study found that the 3-year invasive DFS rate was significantly higher in the olaparib group at 85.9% compared to 77.1% in the placebo group. Similarly, the 3-year distant DFS was 87.5% in the olaparib group versus 80.4% in the placebo group, with both comparisons showing significant HRs favoring olaparib over placebo (an HR of 0.58 and an HR of 0.57, respectively; *p* < 0.001 for both). The safety profile of olaparib was consistent with known side effects, with no excess serious adverse events or adverse events of special interest, including myelodysplastic syndrome and acute leukemia. This robust evidence supports the strategic use of BRCA testing in guiding treatment decisions, aligning with the observed practice trends in BUs that favor testing all eligible patients for potential PARP inhibitor therapy. This approach may ultimately lead to more personalized, effective treatment protocols, enhancing both the quality of life and survival outcomes for patients.

Our survey showed that multi-gene panels are used in 33.0% of cases for all patients initiated to genetic counseling, in 8.3% for all patients potentially eligible for PARP inhibitors treatment, while 56.0% of centers believe the standard criteria for referral to genetic counseling are sufficient. Furthermore, 2.7% of centers report never using multi-gene panels. This selective application contrasts with findings from Fasching PA et al. [27], who evaluated mutations in BRCA1/2 and other cancer predisposition genes in 2595 patients with metastatic BC. They found germline mutations in 12 established BC predisposition genes in 271 patients (10.4%), with BRCA1 or BRCA2 mutations present in 129 patients (5.0%). Notably, BRCA1 mutation carriers had a higher incidence of brain metastases (27.1%) compared to non-carriers (12.8%). The study highlighted that mutations did not significantly affect PFS or overall survival in metastatic BC patients, suggesting that multi-gene panel testing should be considered for all metastatic BC patients due to the high mutation frequency, but also that the clinical utility of finding alterations in genes other than BRCA 1/2 (with the potential exception of PALB2) may be limited. Similarly, the BREAKOUT study [28] reported a 9.7% prevalence of germline BRCA mutations among 341 HER2-negative metastatic BC patients undergoing first-line chemotherapy. Despite not being selected based on germline BRCA mutation risk factors, 5.8% of patients without traditional risk factors still exhibited mutations, supporting guidelines for routine germline BRCA mutation testing in this group. Moreover, Whitworth PW et al. [29] evaluated the impact of universal germline genetic testing on clinical decision-making for BC patients, analyzing 952 patients with significant findings in both in-criteria and out-of-criteria groups according to 2017 National Comprehensive Cancer Network (NCCN) guidelines [30]. One or more changes were reported for 31 of 37 (83.8%) in-criteria patients and 23 of 34 (67.6%) out-of-criteria patients with a pathogenic or likely pathogenic variant. Recommendations were changed as a result of testing results for 14 of 22 (63.6%) out-of-criteria patients who had a variant in a BC predisposition gene. These results demonstrated that universal testing can significantly influence patient management, suggesting that restrictive testing criteria may deny many BC patients the benefits of data-informed treatment modifications, and a broader application of multi-gene panels in clinical practice may enhance personalized treatment strategies and access to targeted therapies. Additionally, Beitsch PD et al. [31] critically evaluated the efficacy of the NCCN guidelines [30] in detecting pathogenic variants in BC patients through expanded panel testing. Over 1,000 BC patients were enrolled in a multicenter prospective registry across 20 sites, utilizing an 80-gene panel test. Their findings highlighted a significant gap in current genetic testing guidelines, with 49.9% of BC patients meeting NCCN criteria and 50.0% not; yet, pathogenic or likely pathogenic variants were identified in 8.6% of the cohort. Interestingly, there was no significant difference in detection rates between those who met the guidelines (9.3%) and those who did not (7.9%). These results underlined a potential underdiagnosis in nearly half of the BC patients who could benefit from expanded multi-gene panel testing. To sum up, previous studies suggest that comprehensive multi-gene panel testing in BC may increase the detection of pathogenic variants, potentially leading to more personalized treatment approaches.

In our survey, we reported that 99.1% of BUs indicated that the presence of a pathogenic BRCA variant influenced loco-regional treatment decisions. This finding is consistent with the literature, which highlights the significant impact of BRCA mutations on the management of BC patients. For instance, a retrospective analysis performed by Ain Q et al. [11] demonstrated that the identification of BRCA mutations significantly impacts clinical management strategies. In their study, the presence of BRCA mutations guided the selection of targeted therapies, such as PARP inhibitors, and influenced surgical decisions, including the choice to perform RRMs. Similarly, a study performed by Edaily S et al. [32] demonstrated that pathogenic BRCA1 and BRCA2 germline variants are associated with distinct clinical management strategies, including risk-reducing surgeries such as mastectomy and salpingo-oophorectomy, the use of platinum-based chemotherapies, and the application of PARP inhibitors for targeted therapy. Our survey results align with the comprehensive review of management strategies for BRCA1/2 mutation carriers, which emphasized the need for personalized treatment plans that consider the specific genetic makeup of each BC patient.

Lastly, to overcome the issues faced by BUs in Italy regarding genetic testing, several strategies can be implemented. Firstly, addressing cost and reimbursement issues through policy changes and increased funding can ensure that genetic testing becomes more accessible and affordable. Organizing the identification process for patients eligible for testing can be achieved by implementing standardized screening protocols and increasing awareness among healthcare providers. Reducing reporting and execution times for pre-test counseling can be managed by investing in advanced laboratory infrastructure and increasing the number of trained genetic counselors. Additionally, enhancing collaboration between multidisciplinary teams can improve the overall efficiency of the genetic testing process.

One of the limitations of this study is the voluntary nature of the survey, which may result in selection bias as the responses might predominantly represent views of those who are particularly engaged or have positive experiences with genetic testing practices, limiting the generalizability of the findings. Another limitation is the reliance on self-reported data, which can introduce subjectivity into the responses and may not accurately reflect all operational realities or adherence to guidelines in practice. Furthermore, the survey did not capture detailed demographic or clinical data from the patients, such as age, specific cancer stages, or other health conditions, which could influence the interpretation of the need and urgency for BRCA testing. Lastly, the survey’s design did not allow for a deep exploration of the reasons behind each BU’s specific practices, which could provide more nuanced insights into the barriers and facilitators of implementing effective genetic testing protocols.

## 5. Conclusions

In conclusion, this survey provides valuable insights into the current practices, challenges, and perspectives related to the therapeutic application of BRCA testing in BUs affiliated with Senonetwork Italia. The results underscore the need for ongoing collaboration and the standardization of practices and address logistical challenges to ensure the optimal integration of BRCA testing into routine clinical care, ultimately contributing to more personalized and effective BC management.

## Figures and Tables

**Table 1:** Logistics and timelines of BRCA testing in Italian breast units.

Questions	Answers (%)
**Q1. Where is your BU located?**	
North	58.7%
Center	23.9%
South/Islands	17.4%
**Q2. Region**	
Abruzzo	1.8%
Basilicata	0.9%
Calabria	1.8%
Campania	3.7%
Emilia-Romagna	9.2%
Friuli-Venezia Giulia	3.7%
Lazio	9.2%
Liguria	1.8%
Lombardy	22.0%
Marche	1.8%
Piedmont	10.1%
Apulia	7.3%
Sicily	3.7%
Tuscany	8.3%
Trentino-South Tyrol	1.8%
Umbria	2.8%
Aosta Valley	0.9%
Veneto	9.2%
**Q3. How many cases of BC does your BU handle yearly?**	
150–200	24.8%
201–300	22.9%
301–400	19.3%
More than 400	33.0%
**Q4. When is the test performed?**	
At diagnosis, or anytimebefore surgery	70.6%
During treatment for early-stage disease	24.8%
At the time of metastatic recurrence	4.6%
**Q5. Are there circumstances in which the test execution has an urgency?**	
Yes	87.2%
No	12.8%
**Q6. Within which timeframe can pre-test counseling be usually planned?**	
Less than 1 week	56.0%
2–3 weeks	34.9%
More than 3 weeks	9.1%
**Q7. Which clinical reporting timelines are guaranteed in case of urgency?**	
Less than 21 days	55.1%
21–40 days	44.0%
40–60 days	0.9%
More than 60 days	0%

Footnotes: BU: breast unit, BC: breast cancer.

**Table 2:** Challenges and practices of BRCA testing in Italian breast units.

Questions	Answers (%)
**Q8. What are the main issues in performing the test at your center? [multiple answers]**	
Cost and reimbursement issues	11.9%
Difficulty in identifying patients to initiate the test	0.9%
Reporting times	35.7%
Execution times for pre-test counseling	13.8%
No issues	46.8%
Other issues	8.3%
**Q9. In BC patients, do you resort to pre-test mini-counseling by a medical specialists of the multidisciplinary team?**	
Yes	78.9%
No	21.1%
**Q10. If ’Yes’ to the previous question—which professional figures normally take care of the mini-counseling? [multiple answers]**	
Medical oncologist	85.3%
Breast surgeon	67.9%
Other	26.6%
**Q11. Which patients with triple-negative BC are usually initiated to the test?**	
All patients	48.6%
Only patients with early-age onset < 36 years	2.8%
<50 years	21.1%
<60 years	27.5%
**Q12. Which HR+ BC patients are usually initiated to the test?**	
Only patients with early-age onset of disease (<36 years), multiple tumors, and/or with a family history suggestive of a hereditary predisposition	89.0%
Other	11.0%
**Q13. With the advent of PARPi in the adjuvant setting, would you consider it appropriate to expand the test execution criteria in HR+ BC patients?**	
Yes, it would be appropriate to test all patients	11.0%
Yes, all patients potentially eligible for PARPi treatment should be tested	86.2%
No, the standard criteria for referral to genetic counseling are sufficient	2.8%
**Q14. Does the finding of a BRCA mutation affect the choice of loco-regional treatment?**	
Yes	99.1%
No	0.9%
**Q15. How frequently do you use multi-gene panels?**	
In all patients initiated to genetic counseling	33.0%
In all patients potentially eligible for PARPi treatment	8.3%
The standard criteria for referral to genetic counseling are sufficient	56.0%
Never	2.7%
**Q16. Do you believe that in the future it will be necessary, for therapeutic purposes, to search for variants on tumor tissue?**	
Yes	93.6%
No	6.4%

Footnotes: BC: breast cancer, HR+: hormone-receptor-positive, PARPi: poly(ADP–ribose) polymerase inhibitor.

## Data Availability

The data presented in this study are available upon request from the corresponding author.

## Supplementary Materials

The following supporting information can be downloaded at: https://www.mdpi.com/article/10.3390/curroncol31070282/s1, Table S1: Senonetwork Italia breast center responders.
