# Supplementary material for: BRCA Testing for Patients Treated in Italy: A National Survey of Breast Centers Associated with Senonetwork
Source: Curr Oncol. 2024 Jun 30;31(7):3815–25. doi: 10.3390/curroncol31070282 (PMC11276301; doi:10.3390/curroncol31070282)
Supplement: Supplementary file 1 [file curroncol-31-00282-s001.zip › curroncol-3071424-supplementary.pdf]

Table S1. Senonetwork Italia breast center responders

Abbonante Francesco	Breast Unit - Azienda Ospedaliera Pugliese Ciaccio - Catanzaro, Italy
Altomare Vittorio	Fondazione Policlinico Universitario Campus Bio-Medico– Roma - Italy
Ambrosiani Luciana	Centro di Senologia Ospedale Valduce - Como, Italy
Andreoli Claudio	Humanitas Mater Domini, Castellanza, Italy
Angiolini Catia	S.O.D. Oncologia della Mammella - Breast Unit- AOU Careggi - Firenze, Italy
Aristei Cynthia	Università degli Studi e Azienda Ospedaliera di Perugia, Italy
Pizzorno Laura	Ospedale Civile San Salvatore - L'Aquila, Italy
Baldissera Antonella	Breast Unit Ospedale Bellaria, AUSL Bologna, Italy
Ballardini Bettina	Breast Unit Multimedica, Gruppo Multimedica S.p.A.- Milano, Italy
Barbero Maggiorino	Ospedale Cardinal Massaia- ASL AT - Asti,Italy
Barellini Leonardo	USL Toscana Nord-Ovest, Ospedale di Livorno, Italy
Battaglia Claudio	Breast Unit ASL1 Imperiese - Ospedale Civile di Sanremo (IM), Italy
Battigelli Luisa	AAS5 Friuli Occidentale - Ospedale di Pordenone, Italy
Savini Agnese	SOD Clinica Oncologica - Azienda Ospedaliero Universitaria delle Marche, Ancona, Italy
Bernini Andrea	Policlinico Universitario Le Scotte – AOU Siena, Italy
Biganzoli Laura	Centro di Senologia, Dipartimento oncologico, Azienda Usl Toscana Centro, Nuovo Ospedale Santo Stefano - Prato, Italy
Biglia Nicoletta †	1. Prof. Ordinario Ginecologia e Ostetricia Università di Torino 2. Struttura Complessa Ostetricia e Ginecologia Ospedale Mauriziano ""Umberto I"" - Torino, Italy"
Bisagni Giancarlo	Azienda USL- IRCCS di Reggio Emilia - Reggio Emilia, Italy
Bortolini Massimiliano	Breast Unit ASL Biella, Nuovo Ospedale degli infermi di Biella, Italy
Bortul Marina	S.S.D. Breast Unit Trieste ASUGI - Trieste, Italy
Buonomo Claudio Oreste	Policlinico Tor Vergata - Roma, Italy
Burelli Paolo	U.O.C. Breast Unit Ospedale "Ca' Foncello" ULSS 2 "Marca Trevigiana" - Treviso, Italy
Burlizzi Stefano	Breast Unit Ospedale "A. Perrino" Brindisi, Italy
Busani Massimo	S.C di Chirurgia Senologica e Breast Unit Asst-Mantova, Italy
Cagossi Katia	Breast Unit AUSL Modena Nord, Carpi (MO), Italy
Cairo Giuseppe	Polo Oncologico "Giovanni Paolo II" P.O. V, Lecce, Italy
Caruso Francesco	HICC Humanitas Istituto Clinico Catanese, Misterbianco (CT), Italy
Cavaliere Francesco	Ospedale Belcolle - ASL Viterbo – Viterbo - Italy
Cedolini Carla	Azienda Sanitaria Universitaria Friuli Centrale - Udine - Italy
Ceribelli Anna	Ospedale San Camillo de Lellis - Rieti, Italy
Cipolla Calogero	AOUP Paolo Giaccone – Palermo, Italy
Ciuffreda Luigi	U.O.C. Chirurgia Senologica e Dipartimento di Scienze Chirurgiche - Breast Unit, IRCCS "Casa Sollievo della Sofferenza" Opera di San Pio da Pietrelcina, San Giovanni Rotondo (FG), Italy
Corsi Fabio	1. Facoltà Medicina e Chirurgia, Dipartimento di Scienze Biomediche, Università degli Studi di Milano - Milano, Italy

Table S1. Senonetwork Italia breast center responders

	2. Breast Unit, Unita Operativa di Chirurgica - Istituti Clinici Scientifici Maugeri IRCCS - Pavia, Italy "
Cramarossa Monica	O.C. S.S. Annunziata -Taranto - Breast Unit -ASL TA - Taranto
Custodero Olindo	Breast Unit P.O. San Paolo ASL BA – Bari, Italy
De Laurentiis Michelino	Dipartimento di Oncologia Senologica - Istituto Nazionale Tumori “Fondazione PASCALE” - Napoli - Italy
De Luca Antonio	Breast Unit - ASST Grande Ospedale Metropolitano Niguarda - Milano, Italy
De Vita Roy	Istituto Nazionale dei Tumori Regina Elena - Roma, Italy
Defilippi Loredana	S.S. Senologia Chirurgica, ASLCN2 ALBA-BRA, Ospedale Michele e Pietro Ferrero - Verduno (CN) , Italy
Del Mastro Lucia	Policlinico San Martino IRCCS Università Genova - Genova, Italy
Delle Fratte Franca	ASL Roma 2 Ospedale S.Pertini e Ospedale Sant'Eugenio Roma, Italy
Di Marzio Enrico	UOC di Chirurgia Senologica dell'Aulss7, Ospedale Santorso Alto Vicentino e Bassano del Grappa (VI), Italy
Di Millo Marcello	Breast Unit - Policlinico Foggia, Italy
Di Santo Pina	UOC Chirurgia Senologica AOR San Carlo, Potenza, Italy
Falcini Fabio	Azienda AUSL della Romagna - Forlì (FC), Italy
Ferro Antonella	Breast Unit Ospedale Santa Chiara/APSS, Trento, Italy
MastropietroTiziana	Azienda Ospedaliera San Giovanni-Addolorata, Roma, Italy
Francesconi Duilio	U.O. di Senologia Ospedale Versilia - Lido Di Camaiore (LU), Italy
Fregoni Vittorio	B.U. ASST Valtellina e dell’Alto Lario - Sondalo (SO), Italy
Frittelli Patrizia	Ospedale Fatebenefratelli Isola Tiberina Gemelli Isola – Roma,Italy
Furci Marco	U.O.S.D. "Breast Unit" - Policlinico "G. Rodolico - San Marco" - Catania, Italy
Garrone Ornella	Fondazione IRCCS Ca Granda Ospedale Maggiore Policlinico, Milano, Italy
Generali Daniele	Azienda Socio Sanitaria Territoriale di Cremona, Italy
Gentilini Oreste Davide	IRCCS Ospedale San Raffaele, Milano, Italy
Giordano Monica	Unità Operativa Oncologia ASST – Lariana - Como, Italy
Giotta Francesco	IRCCS Istituto Tumori "Giovanni Paolo II" - Bari, Italy
Giovanazzi Riccardo	Chirurgia Senologica - Breast Unit - IRCCS San Gerardo dei Tintori - Monza (MB), Italy
Grassi Massimo Maria	Unità Operativa di Senologia, Humanitas Gavazzeni, Bergamo, Italy
Grossi Simona	U.O.C. Chirurgia Generale a indirizzo Senologico - EUSOMA Centre ASL02 Abruzzo - Ortona (CH), Italy
Huscher Alessandra	Breast Unit - Fondazione Poliambulanza - Brescia, Italy
Lamanna Ginevra	Ospedale SS. Annunziata Savigliano-Cuneo, Italy
Lolli Gianfranco	S.S.D. Chirurgia Senologica - Nuovo Ospedale San Giovanni Battista - Foligno (PG), Italy
Magni Carla	S.C. Breast Unit ASST Lecco, Italy
Malossi Alessandra	SC Oncologia ed Ematologia Oncologica, Ospedale U. Parini - Aosta, Italy
Mancini Stefano	Chirurgia Generale, Ospedale Universitario Luigi Sacco, Università degli Studi di Milano - Milano, Italy
Marano Antonio	Chirurgia Senologica, Ospedale del Mare - Napoli, Italy
Marenco Davide	ASLTO5 - Ospedale Santa Croce - Moncalieri (TO), Italy

Table S1. Senonetwork Italia breast center responders

Massarut Samuele	Centro di Riferimento Oncologico di Aviano (CRO Aviano), IRCCS - Aviano (PD), Italy
Massocco Alberto	Chirurgia Senologica, IRCCS Ospedale Sacro Cuore Don Calabria - Negrar di Valpolicella (VR), Italy
Meggiolaro Fabrizio	U.O.S. Chirurgia Senologica, Azienda Ospedale-Università Padova, Italy
Melucci Giuseppe	Breast Unit di Rimini-Santarcangelo – Rimini, Italy
Meneghini Graziano	U.O.C. Chirurgia Senologica AULSS n. 8 Berica - Vicenza, Italy
Millo Francesco	S.S.D. Breast Unit ASL AL Ospedale "SS Antonio e Margherita" - Tortona (AL), Italy
Mirri Maria Alessandra	UOC di Radioterapia della ASL Roma1 - Dipartimento Oncologico ASL Roma 1 - Breast Unit ASL Roma1 - Roma, Italy
Montemezzi Stefania	Azienda Ospedaliera Universitaria Integrata di Verona, Verona, Italy
Moschetta Marco	Dipartimento Interdisciplinare di Medicina (DIM) - Breast Care Unit Facoltà di Medicina - Università di Bari – Bari, Italy
Musolino Antonio	Breast Unit Interaziendale della Provincia di Parma - Azienda Ospedaliero Universitaria di Parma - Parma, Italy
Palli Dante	U.O.C. Chirurgia Generale ad Indirizzo Senologico AUSL Piacenza - Piacenza, Italy
Papaccio Guido	Azienda Ulss 3 Serenissima Venezia - Mestre (VE), Italy
Pieraccini Mariagrazia	Ospedale della Misericordia Grosseto, Italy
Polato Romano	Brest Unit - Ospedale di Bolzano , Italy
Renne Maria	Breast Unit A.O.U. Mater Domini - Catanzaro, Italy
Ricci Fabio	Breast Unit, Ospedale Santa Maria Goretti - Latina, Italy
Ridolfo Raffaella	Breast Unit, Azienda Sanitaria Territoriale Ancona (AST AN), Italy
Ristagno Maurizio	Vittorio Emanuele Gela Breat Unit, Caltanissetta, Italy
Romanucci Giovanna	Breast Center AULSS 9 Scaligera – Verona, Italy
Roncella Manuela	Centro Senologia, Azienda Ospedaliero Universitaria Pisana, Italy
Rossi Lorenzo	Centro di Senologia della Svizzera Italiana – Ente Ospedaliero Cantonale. Viganello – Lugano,Switzerland
Roveda Laura	Ospedale 'F. Lotti' - Pontedera, USL Toscana Nordovest - Italy
Rovera Francesca Angela	ASST Settelaghi - Università degli Studi dell'Insubria - Varese, Italy
Sanguinetti Alessandro	Azienda Ospedaliera Terni, Italy
Santoriello Antonio	Casa di Cura "Prof. Dott. Luigi Cobellis", Vallo della Lucania (SA), Italy
Sciamannini Maria	S.O.S. Chirurgia Senologica, Breast Unit Ospedale San Jacopo - Pistoia, Italy
Anghelone Chiara Annunziata Pasqualina	S.C. Chirurgia Generale 3-Senologia e SSD Chirurgia dei Tumori Eredo-famigliari, Fondazione IRCCS Policlinico San Matteo - Pavia, Pavia, Italy
Simoncini Edda	Azienda Spedali Civili Brescia/SSVD Breast, Brescia, Italy
Stancampiano Pietra	Struttura Semplice di Senologia ASLVCO - Verbania (VB) , Italy
Steffano Giovanni Battista	Breast Unit - Policlinico di Monza - Monza, Italy
Taffurelli Mario	Università degli studi di Bologna - Ospedale S. Maria della Scaletta – Imola, Italy
PiacentiniFederico	Breast Unit A.O.U. Policlinico di Modena, Italy

Table S1. Senonetwork Italia breast center responders

Tinterri Corrado	Breast Unit, IRCCS Humanitas Research Hospital, Via Manzoni 56, 20089 Rozzano, Milan, Italy
Toesca Antonio	Candiolo Cancer Institute, FPO - IRCCS, Candiolo (TO), Italy
Pegoraro Maria Cristina	Centro di Senologia Ospedale Pederzoli - Peschiera del Garda (VR), Italy
Trunfio Martino	UOC Chirurgia SenologicaAORN A. Cardarelli -Napoli, Italy
Ucci Giovanni	Azienda Socio Sanitaria Territoriale di Lodi –Lodi, Italy
Valieri Luca	AULSS 5 Polesana, UOS Chirurgia senologica Ospedale Santa Maria della Misericordia - Rovigo, Italy
Vandone Anna Maria	A.O S. Croce a Carle - Breast Unit - Cuneo, Italy
Veronesi Paolo	1. Divisione Senologia Chirurgica, Istituto Europeo di Oncologia, IRCCS - Milano, Italy<br>2. Dipartimento di Emato-Oncologia, Università degli Studi di Milano, Italy
Zamagni Claudio	Breast Center, IRCCS Azienda Ospedaliero-Universitaria di Bologna Policlinico di Sant'Orsola - Bologna, Italy
Zanon Eugenio	Breast Unit - Ospedale Cottolengo - Torino, Italy
